# Social networks predict selective observation and information spread in ravens

**DOI:** 10.1098/rsos.160256

**Published:** 2016-07-13

**Authors:** Ipek G. Kulahci, Daniel I. Rubenstein, Thomas Bugnyar, William Hoppitt, Nace Mikus, Christine Schwab

**Affiliations:** 1 Department of Ecology and Evolutionary Biology, Princeton University, Princeton, NJ 08544, USA; 2 Department of Cognitive Biology, University of Vienna, Althanstrasse 14, 1090 Vienna, Austria; 3 School of Biology, Leeds University, Leeds, UK; 4 Messerli Research Institute, University of Veterinary Medicine Vienna, Medical University of Vienna and University of Vienna, 1210 Vienna, Austria

**Keywords:** information transmission, social networks, observation networks, network-based diffusion analysis, order of acquisition diffusion analysis, *Corvus corax*

## Abstract

Animals are predicted to selectively observe and learn from the conspecifics with whom they share social connections. Yet, hardly anything is known about the role of different connections in observation and learning. To address the relationships between social connections, observation and learning, we investigated transmission of information in two raven (*Corvus corax*) groups. First, we quantified social connections in each group by constructing networks on affiliative interactions, aggressive interactions and proximity. We then seeded novel information by training one group member on a novel task and allowing others to observe. In each group, an observation network based on who observed whose task-solving behaviour was strongly correlated with networks based on affiliative interactions and proximity. Ravens with high social centrality (strength, eigenvector, information centrality) in the affiliative interaction network were also central in the observation network, possibly as a result of solving the task sooner. Network-based diffusion analysis revealed that the order that ravens first solved the task was best predicted by connections in the affiliative interaction network in a group of subadult ravens, and by social rank and kinship (which influenced affiliative interactions) in a group of juvenile ravens. Our results demonstrate that not all social connections are equally effective at predicting the patterns of selective observation and information transmission.

## 1. Introduction

Individual variation in traits such as social rank, motivation and personality can result in some individuals acquiring novel information sooner and/or more accurately than others [1–4]. Such variation in information acquisition introduces opportunities for conspecifics to observe and learn from each other, resulting in social transmission where novel behaviour spreads from one individual to another [5]. For instance, when faced with a novel task, naive individuals can acquire information about the task solution by observing informed group members, before using this information to solve the task themselves. However, social transmission rarely happens at random. Instead, animals are frequently selective in which informed conspecifics' behaviour they observe. For example, vervet monkeys preferentially observe and acquire information from the behaviour of females [6], ravens use information from their kin when they are in groups of same-aged conspecifics [7], chimpanzees acquire information by observing older and/or dominant group members [8,9] and domestic fowl use information from dominant conspecifics [10]. The social connections between conspecifics can also influence who observes whom and who learns from whom [11]. Group members frequently interact with each other in multiple social contexts that range from affiliative interactions to aggressive interactions [12–16]. The presence and the frequency of social connections in one or more of these contexts may drive selectivity in who observes whom, eventually resulting in animals acquiring and using information from the conspecifics to whom they are socially connected. Yet, hardly anything is known about the effectiveness of different social connections in reliably predicting the patterns of informationtransmission.

Here, we analyse the relationships between social connections, selective observation and learning patterns to investigate the role of different social contexts in information transmission. Social network analysis provides a powerful tool to quantify social connections in multiple contexts [13,17]. Use of network models such as network-based diffusion analysis (NBDA), which infer social transmission of a novel behaviour when its pattern of diffusion follows a social network [18], makes it possible to analyse the role of network connections in information transmission. A variant of NBDA, order of acquisition diffusion analysis (OADA), analyses the temporal order with which different individuals perform a novel behaviour [4]. NBDA and OADA integrate networks with learning experiments [12,14,19–24] and have been used to explore transmission of tool use in chimpanzees [24], lobtail-feeding technique in whales [20], foraging traditions in tit species [21,23], latency of novel task discovery (but not task solving) in fish [12] and patch discovery through cross-species association networks in mixed-species flocks [22]. However, whether or not different types of social connections, such as affiliative and agonistic interactions, influence information transmission to varying extents has not yet been tested.

Inferences about group transmission can only be made when naive individuals have the option of choosing which informed conspecifics to observe and learn from [25,26]. Attending to others' behaviour can play a significant role in transmission if observation influences future behaviour [11]. Yet, network analyses have rarely been used to quantify selectivity in attention during information transmission. To address whether naive individuals selectively attend to specific informed group members, an observation network based on who observes whom in the presence of novel information can be constructed and analysed in relation to networks based on social connections. Finding that the same social network correlates with both the observation network and the order with which different individuals learn a novel behaviour would provide strong evidence for the role of that social context in information transmission.

To determine which social connections predict selective observation and information transmission, we worked with two common raven (*Corvus corax*) groups. Ravens are renowned for paying attention to [27] and learning from each other [7,28]. Adult ravens are pair-bonded and territorial [29], but non-breeding ravens form fission–fusion groups in which they build strong relationships with some of their conspecifics [30,31]. In each group, we constructed three social networks on affiliative interactions, agonistic interactions and physical proximity. We then seeded novel information, first by isolating and training one female from each group on a foraging task, and then allowing those females to perform the solution to their respective groups. We constructed an observation network in each group, based on which naive individuals observed which informed conspecifics' task-solving behaviours. These observation networks were then used to determine who acquired task-solving information from whose behaviour, before using this information to solve the task.

Affiliative interactions, such as allo-preening (or allo-grooming) and food sharing, are considered reliable indicators of strong social bonds in multiple taxa [32–35]. If acquiring information about a novel problem requires multiple observations from a close distance, then individuals would be more likely to observe their affiliates with whom they share social bonds, as these bonds would increase their tolerance for each other in close proximity. When tested in dyads, ravens pay more attention to the behaviour of their affiliates than their non-affiliates [36]. A similar selectivity may exist in a group, leading to ravens selectively attending to and acquiring information from their affiliates. This would result in correlations between the networks based on positive social connections, such as affiliative interaction and physical proximity networks, and both the observation network and the order in which the task solution is performed by group members. Thus, we predicted that networks based on positive social connections will influence the patterns of selective observation and information transmission. We used three complementary approaches to test this prediction.

First, we analysed whether naive individuals selectively observe the task-solving behaviour of the informed conspecifics with whom they share positive social connections. We used network regression analysis to determine whether the connections in the affiliative interaction network and in the proximity network predict the connections in the observation network. Second, we investigated whether socially central individuals solve the task sooner than others. Central individuals are well connected to their group members and are thus more likely to be connected to at least one conspecific who has already solved the task. Being socially central is advantageous for learning from others, especially if individuals preferentially acquire and use information from the conspecifics with whom they share social connections. We predicted that ravens with high social centrality in affiliative interaction and proximity networks will solve the task sooner than their less central conspecifics, providing further evidence that positive social connections are influential in observation and transmission. Finally, we used OADA to determine whether networks based on positive social connections reliably predict information transmission. If this is the case, then the naive individuals who are connected to informed group members in the affiliative interaction and proximity networks should learn the solution sooner than those who are not connected to informed group members.

## 2. Material and methods

### 2.1. Social network data collection

We studied two captive raven groups at the Haidlhof Research Station, an outdoor laboratory of University of Vienna and of University of Veterinary Medicine, Vienna in Austria. One group included 12 subadult ravens (2–3 years old at the time of testing; seven females, five males; electronic supplementary material, table S1). The second group included 10 juveniles (less than 1 year old; three females, seven males; electronic supplementary material, table S1). Relatedness differed between these two groups; 9 of 10 juveniles had at least one sibling in their group, while only 4 of 12 subadults had a sibling. Non-breeding ravens form fission–fusion groups in the wild [30,31] where they frequently face changing group dynamics. Working with groups that varied in age and kinship allowed us to account for the role that group composition differences, such as variation in age and relatedness, plays in information transmission. The two groups were housed separately from each other in four connected outdoor enclosures (10 × 18 m), each of which featured indoor compartments and enrichment with branches, twigs and stones. Both groups were fed twice a day and had ad libitum access to water. All ravens were marked with unique colour bands and were habituated to the experimenter (I.G.K.).

In each group, we collected social data with a handheld HD camcorder from outside the enclosures. These observational sessions were conducted for a minimum of 20 min per day for 98 days between September 2012 and February 2013, excluding the days on which task experiments were in session (13 January–10 February in subadults; 3–10 February in juveniles). The identity and the location of the ravens were narrated to the videos. We used all-occurrence sampling to collect affiliative and agonistic interaction data, and scan sampling (every 15 min) to collect proximity data [37]. Affiliative interactions included two measures: physical contact (allo-preening, touching with feet and beak-to-beak contact) and sharing (manipulating food or objects within 1 m of each other, which indicates tolerance of each other in the presence of food or objects). Agonistic interactions included fights, chases and retreats after receiving threats. Physical proximity data also included two measures: sitting close and sitting on the same branch. Sitting close was defined as two individuals perched close enough to make physical contact with each other without moving, but not actually interacting with each other. Sitting on the same branch was defined as perching on the same branch (branches were 2–4 m in length) and excluded the ravens who were sitting close to each other. If three ravens (A,B,C) were sitting in that order, close enough to make contact with their immediate neighbour, then A–B and B–C were considered sitting close, but A–C were considered to be sitting on the same branch.

### 2.2. Task trials

**Figure 1.**
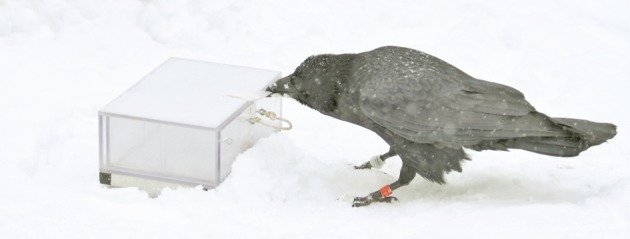
Photo of a raven opening the Velcro (also see electronic supplementary material, videos S1 and S2).

We used an artificial foraging task (clear Plexiglas box; 30 cm (l) × 12 cm (h) × 20 cm (w)) as novel information. The task required solving two steps, first by opening a Velcro strip holding a drawer shut, and subsequently by pulling a string to open the drawer (figure 1; electronic supplementary material, videos S1 and S2). We chose a female from each group and trained her on the task solution in a separate compartment that was out of sight of other ravens. These two females were chosen based on the results of previous experiments which showed that they were more likely than others to approach novel objects and solve cognitive tasks. Each of the training sessions lasted either for 30 min or until the female did not approach the task for 10 min. The subadult female first solved the task after three training sessions, while the juvenile female first solved it after six training sessions. Both females were able to solve the task consistently during the rest of the training sessions after having solved it once.

We began the group testing phase in each group after their trained female had solved the task 10 consecutive times. All ravens were familiar with how to open the drawer from previous experiments. However, only the trained females had experience with the Velcro. Thus, we focused on Velcro learning for assessing information transmission. During the group testing phase, we placed the whole group in a single compartment to allow all conspecifics to see the task solution. The task was presented for 30 min sessions. No more than three sessions were run per day in each group. Subadults required 27 sessions for all individuals to solve the task, while juveniles required 16 sessions. Each refill was considered a trial, and each session consisted of multiple trials (mean ± s.d. = 13.3 ± 5 trials in subadults, 14.9 ± 5 in juveniles). We used only one piece of reward (Frolic dog food) per trial to minimize scrounging. We placed the task in an open area, where group members could see it without branches blocking their view, but such that ravens in the other group could not see it. To minimize disturbances during task refilling, we filled the task on the spot by lifting it from the ground and blocking ravens' view of it. Each raven was free either to participate in the experiment (by observing or by contacting the task) or to move away from the experiment. No data on proximity or social interactions were collected during these sessions, nor on the days during which trials were run, to prevent the task presence from influencing the social connection data.

From the trial videos, we noted the identity of the ravens who (i) contacted the task on any part except Velcro, (ii) contacted the Velcro but did not open it (unsuccessful manipulation), (iii) successfully opened the Velcro, the criteria by which we defined task solution and learning (ravens were familiar with how to open the drawer from previous experiments), (iv) took the reward, (v) observed another raven solve the task. Observing was defined as being within 1 m radius of the task while another raven opened it. This definition identified observation as attentiveness to task solution from close proximity. We chose 1 m as our cut-off for observing because multiple ravens were frequently around the task while it was solved (electronic supplementary material, video S2), and their presence may have prevented those who were farther than 1 m from seeing the solving technique.

During the last sessions in each group (last three sessions in subadults, last two in juveniles), we moved the ravens who had solved the task out of the testing enclosure, to present the task only to those who had not yet solved it. During these sessions, non-solvers from each group were tested together (one subadult female and two subadult males were tested together, two juvenile males were tested together; electronic supplementary material, table S1). Although aggressive interactions such as physical fights rarely happened around the task, subordinates were sometimes displaced by more dominant conspecifics. Testing non-solvers allowed us to determine whether they had acquired information about the task solution during their observations, but did not solve due to competition or social interference. We separated these individuals only at the end of the trials in both groups, after the rest of their group members had solved the task, to minimize the effect that the separation may have on the overall transmission patterns.

### 2.3. Network analysis

Social data were converted into network matrices and analysed in UCINET (v. 6.507) [38]. We calculated three network measures (strength, eigenvector centrality, information centrality), each of which quantifies a different aspect of social centrality, and ranked each raven's measures from each network relative to their group members' measures. *Strength*, also known as weighted degree, defines the frequency of connections between pairs. Degree indicates how many individuals each group member is connected to, while strength indicates how frequently each of those connections happen. We used Freeman's degree centrality in UCINET [39] to calculate strength from weighted and directed networks. Directed networks (e.g. affiliative and agonistic interaction networks) include a separate actor and a receiver. In these networks, *out-strength* (weighted out-degree) indicates the frequency of interactions that an individual initiates, while *in-strength* (weighted in-degree) indicates the frequency of interactions that an individual receives. *Eigenvector centrality* provides insight into the centrality of an individual based on the centrality of those to whom it is connected. *Information centrality* is useful in determining the amount of information that can be transmitted in the network, by accounting for each network connection that can potentially reach a particular individual [40]. We analysed networks as weighted networks, when possible, to preserve information about the strength of the interactions. Weighted networks are especially useful in captive groups and in small groups where the frequency of connections is more informative than their presence [41,42].

We constructed an observation network based on who observed whom during task solving. Thus, in each group, we ended up with four distinct networks (affiliative interactions, agonistic interactions, proximity and observation). Observation networks included only directed (non-reciprocal) connections, because observation data were obtained only from the naive ravens before they solved the task for the first time. Thus, in our observation networks, a naive raven who observed an informed conspecific was never observed by that particular conspecific. This allowed us to include only the observations that contributed to the first task-solving event for each individual. We then normalized the observation networks because some ravens had solved the task more frequently than others did. For example, if A solved the task X times before B first solved it, and B observed A for Y times before solving it for the first time, then Y/X was entered to the cell corresponding to B observing A.

Using Multiple Regression Quadratic Assignment Procedure (MRQAP, double Dekker semipartialling variant) in UCINET [43] in each group, we analysed which factors predicted the connections in the observation networks. The dependent variable was the observation network, and the independent variables were the networks on affiliative interactions, agonistic interactions, proximity, sex similarity (1 for same sex, 0 for different sexes) and (relative) similarity in social rank. Social rank was calculated from a linear hierarchy based on retreats after receiving a threat (MatMan 1.1, I&SI method, Noldus Information Technology) [44,45]. MRQAP has previously been used to analyse the relationships between networks in multiple species [46–51]. It first runs a regression test for the corresponding cells of each matrix, and then permutes the rows and the columns of the dependent matrix to repeat this regression multiple times (we ran 10 000 permutations) [38,52].

### 2.4. Task-solving order analysis

To determine whether ravens with high social centrality solved the task sooner and thus had high centrality in the observation network, we used the non-parametric Spearman's rank correlation test on the ranked centrality measures. We ran two analyses using Spearman's test. First, we analysed the correlations between the ranked centrality measures from the social networks (affiliative interaction, agonistic interaction, proximity) and the task-solving order. Second, we analysed the correlations between the ranked centrality measures from the social networks and the observation networks. For this second analysis, only the same measures were compared with each other (e.g. instrength in affiliative network was compared only to instrength in observation network). The trained females were excluded from the rank correlation analyses. If ravens with high social centrality are observed more frequently and/or by more individuals, this would suggest that they act as important information sources during information transmission.

We used the OADA variant of the NBDA to determine the predictive power of different networks [22]. We analysed which social networks (affiliative interactions, agonistic interactions, proximity) predict the order with which ravens perform the task solution for the first time. Note that we did not include observation networks in OADA. OADA assumes that the rate of transmission from an informed individual (*j*) to a naive individual (*i*) is proportional to the network connection between them (*a_*ij*_*). However, the model can be expanded such that the rate is proportional to *a_*ij*_* × *w_*j*_*, where *w_*j*_* is the transmission weight reflecting the total number of times (*j*) solves the task. Models with transmission weights are based on the assumption that transmission is proportional to the rate at which the task solution is performed by an informed conspecific. Models without transmission weights assume that all informed conspecifics transmit the task solution at the same rate regardless of how often they solve the task themselves. We fitted models both with and without transmission weights. Sex and social rank in both groups, as well as kinship in juveniles, were included as variables that potentially influence the task-solving order. We used an information theoretic approach, using corrected Akaike's information criteria (AIC_c_), to account for model selection uncertainty and to assess the support for each network relative to models based on asocial learning (models based on asocial learning included sex and social rank; see the electronic supplementary methods for model details).

## 3. Results

### 3.1. Observing conspecifics' task-solving behaviour attracts ravens' attention to the task

All ravens (*n* = 22) participated in the experiments, and all except one subadult male solved the task by opening the Velcro strip before pulling the drawer to access the reward. Most ravens, except the two juveniles who were tested separately from their group in the last two sessions, observed at least one group member within 1 m radius of the task before solving it for the first time (number of task-solving events observed before solving, subadults: 20.09 ± 32, juveniles: 17.78 ± 17.9; number of conspecifics observed before solving, subadults: 3.63 ± 2, juveniles: 2.55 ± 2.1). Before solving the task for the first time, each raven contacted the task at least once by pecking at it or by pulling on the string (mean ± s.d. of contacts before solving the task, subadults: 12.72 ± 13.9, juveniles: 2.89 ± 1.6; electronic supplementary material, table S1). Most contact occurred on places other than Velcro (total number of contacts before solving, subadults: 140, juveniles: 26; contacts on Velcro, subadults: 1, juveniles: 5). The five contacts on Velcro by naive juveniles were extremely brief, because they got displaced by a more dominant conspecific soon after contacting the Velcro. Overall, ravens were more likely to contact the task after having observed a conspecific within a 1 m radius in previous trials (number of contacts after observing, subadults: 12.55 ± 14, juveniles: 2.44 ± 2; number of contacts before observing, subadults: 0.44 ± 0.7, juveniles: 0.18 ± 0.4; electronic supplementary material, table S1). Regardless of social rank or sex, ravens who contacted the task frequently had also observed frequently (multiple regression: *F*_3,19_ = 5.039, *p* = 0.012 for the whole model; effect of observing frequency on contact frequency: *F* = 14.219, *p* = 0.002; effect of social rank: *F* = 0.108, *p* = 0.746; effect of sex: *F* = 0.228, *p* = 0.639), suggesting that observing others attracted ravens' attention to the task.

### 3.2. Ravens observe their affiliates

**Figure 2.**
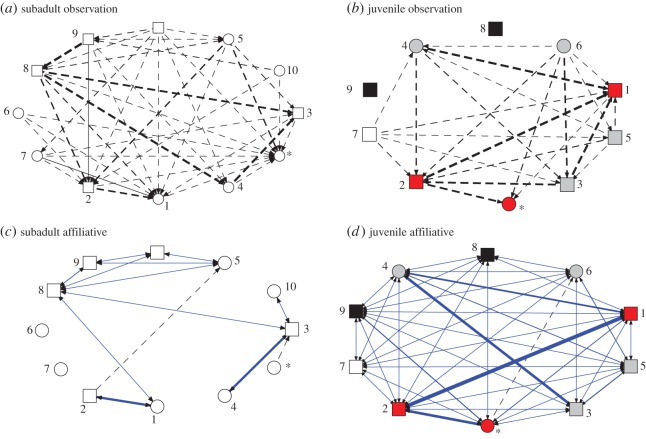
Observation and affiliative interaction networks. Arrows in the observation networks (*a*,*b*) indicate gaze direction of naive ravens, and point towards the informed ravens whose task-solving behaviour they observed. Arrows in the affiliative interaction networks (*c*,*d*) point towards the recipient of the affiliative interaction. Circles represent females, rectangles represent males. Line thickness is proportional to the connection frequency (strength). Blue solid lines indicate reciprocal connections, black dashed lines indicate non-reciprocal connections. All connections in the observation network are non-reciprocal; the observation network includes only the observations made by naive ravens before they solved the task for the first time. Numbers indicate task-solving order, asterisks indicate the trained females. Same coloured nodes in juveniles (*b*,*d*) represent kin groups. The nodes are spatially arranged in a circle layout, based on ID, for the ease of comparison between networks.

We calculated the density of the networks to determine whether ravens were selective in their social connections and in their observations. A network based on high social selectivity has low density, which suggests that the majority of connections that could potentially exist in the network do not actually exist. Subadults were more selective in their social connections than juveniles were (affiliative network density in subadults: 0.182, in juveniles: 0.877, proximity network density in subadults: 0.409, in juveniles: 0.911; agonistic network density in subadults: 0.576, in juveniles: 0.656). Observation networks had low density in both groups (subadults: 0.303, juveniles: 0.256, figure 2*a*,*b*), suggesting that ravens were highly selective in whom they observed. To determine which factors influenced selectivity in who observed whom, we used the MRQAP analysis. MRQAP revealed that ravens selectively observed the group members towards whom they initiated frequent affiliative interactions, or to whom they frequently perched in close proximity (MRQAP, table 1 and figure 2*c*,*d*). Observation did not depend on homophily; ravens were not more likely to observe the same-sex conspecifics or those with similar social rank to themselves (table 1). Proximity and social interaction data were collected only on the days when the task trials were not in session, allowing us to reliably separate proximity and interaction networks from the observation networks. Overall, ravens' decision of whom to observe was determined mainly by the socio-positive behaviours such as affiliative interactions and tolerance of close proximity.

**Table 1.** Multiple regression quadratic assignment procedure (MRQAP) results. Results in italics indicate a significant effect of the respective independent variable on the dependent variable (the observation network).

group	independent variable	coefficient	s.e.	*p*-value
subadults (*n* = 12)	affiliative interaction	0.478	0.335	*0*.*012*
	agonistic interaction	−0.106	0.338	0.241
	proximity	−0.192	0.469	*0*.*019*
	social rank	0.088	0.383	0.223
	sex	<−0.001	2.326	0.496
juveniles (*n* = 10)	affiliative interaction	0.896	0.483	*<0*.*001*
	agonistic interaction	−0.129	0.677	0.212
	proximity	−0.558	0.584	*0*.*008*
	social rank	−0.149	1.038	0.202
	sex	0.037	5.132	0.328

### 3.3. Ravens with high affiliative network centrality play important roles in transmission

**Figure 3.**
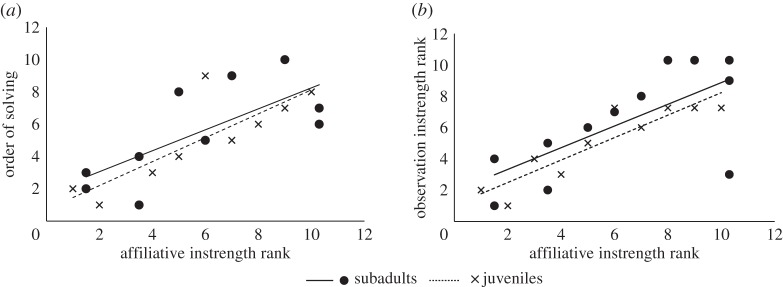
Task solving, observation and affiliative interactions. Instrength rank in the affiliative network is correlated with task-solving order (*a*) and with instrength rank in the observation network (*b*). Lower rank numbers indicate higher centrality.

To address whether socially central ravens solved the task sooner, we ranked each individual's centrality measures (strength, eigenvector, information centrality) in each network relative to their group member's measures (electronic supplementary material, table S2). In both groups, the majority of the centrality measures from the affiliative interaction network correlated with the task-solving order. In particular, ravens who solved the task sooner had initiated and received frequent affiliative interactions (Spearman's rank correlation between task-solving order and affiliative network measures in subadults: out-strength *r* = 0.72, *p* = 0.019, in-strength *r* = 0.722, *p*= 0.018; in juveniles: out-strength *r* = 0.85, *p* = 0.004, in-strength *r* = 0.817, *p* = 0.007, figure 3*a*). Juveniles who solved the task sooner had high information and eigenvector centrality in the affiliative network (juveniles' information centrality: *r* = 0.817, *p* = 0.007, eigenvector centrality: *r* = 0.800, *p* = 0.010; subadults' information centrality: *r* = 0.073, *p* = 0.841, eigenvector centrality: *r* = 0.491, *p* = 0.149). Individuals with high affiliative network centrality were observed more by others and had high centrality in the observation network (Spearman's rank correlation between affiliative and observation instrength in subadults: *r* = 0.924, *p* < 0.001; in juveniles: *r* = 0.897, *p* < 0.001, figure 3*b*; between affiliative and observation information centrality in subadults: *r* = 0.838, *p* = 0.001; in juveniles: *r* = 0.854, *p* = 0.003). The majority of the centrality measures from the agonistic interaction network and the proximity network were not correlated with the task-solving order nor with the observation network centrality measures in either group (electronic supplementary material, table S3). Overall, ravens with high centrality in the affiliative interaction network solved the task sooner and were central in the observation network as a result of being observed more by naive conspecifics.

### 3.4. Transmission of task solving in subadults

Using OADA, we calculated the support that each network (affiliative interactions, agonistic interactions, proximity) provided for social transmission relative to models based on asocial learning. We first calculated the Akaike weight for each model we fitted [53], and then obtained the relative support for each network, by summing over all the models that included that particular network. We also calculated the support for the asocial models by obtaining summed Akaike weights for these models. We then obtained a ‘support ratio’ by dividing the support for each network by the support for the asocial models. Support ratio thus indicates the support that each network provides for social transmission relative to asocial learning (see the electronic supplementary methods for details). The strength of support ratios can be interpreted, as a guideline, such that a *p*-value of 5% in a likelihood ratio test between two models that differ in one parameter (e.g. social transmission via one network) would correspond to a support ratio of 2.5.

The affiliative network with transmission weights provided the most support for social transmission against asocial learning in subadults. The support ratio for the affiliative network with transmission weights was 2.24, meaning that there was 2.24 times more support for social transmission following this network than there was support for asocial learning (table 2). The affiliative network was composed of two behaviours: physical contact (such as allo-preening) and sharing. We analysed these two components separately to explore which one contributed to the observed patterns of transmission. Running OADA separately on physical contact and on sharing revealed that the physical contact component provided the main support for social transmission (support ratio for physical contact = 3.42; support ratio for share = 1.64). Neither the agonistic interaction network nor the proximity network provided support in subadults (support ratio for agonistic interaction = 0.41; support ratio for proximity= 1.73). Even when we separated the proximity network into its two components (sitting close and sitting on the same branch), as we had done with the affiliative interaction network, we did not find support for transmission (support ratio for sitting close = 1.76; support ratio for sitting on the same branch = 1.07). Furthermore, there was no support for the effect of social rank (total Akaike weight for social rank =  37.29%) and weak support for the effect of sex (total Akaike weight for sex = 54.87%). Overall, the affiliative interaction network was the best predictor for transmission in subadults.

**Table 2.** Order of acquisition diffusion analysis (OADA) results. Support ratios for each network are shown. Results in italics indicate the networks with a support ratio of greater than 2 (i.e. networks that provide at least 2× more support for social transmission relative to asocial learning). For the affiliative interaction and the proximity networks, we also provide the support ratios for the specific behaviours that make up each of these two networks.

group	network	with weights	without weights
subadults (*n* = 12)	affiliative interaction	*2*.*24*	1.31
	agonistic interaction	0.41	0.62
	proximity	1.73	1.43
	physical contact (affiliative)	*3*.*42*	*2*.*6*
	share (affiliative)	1.64	1.09
	sitting close (proximity)	1.76	1.03
	same branch (proximity)	1.07	1.14
juveniles (*n* = 10)	affiliative interaction	*6*.*82*	*4*.*47*
	agonistic interaction	0.16	0.19
	proximity	*6*.*85*	*10*.*79*
	physical contact (affiliative)	0.05	0.16
	share (affiliative)	*13*.*94*	*7*.*94*
	sitting close (proximity)	*2*.*54*	*61*.*48*
	same branch (proximity)	*10*.*62*	*17*.*12*

For the physical contact component of the affiliative network, which provided the main support for social transmission, we calculated the social transmission parameter (*s*) to estimate the rate of social transmission, relative to asocial learning, per unit connection (i.e. transmission between two individuals with connection = 1 and transmission weight = 1). The social transmission parameter (*s*) was 7.76 (95% CI = [5.43, 2100.19]), meaning that a naive raven, who had a single connection of 1 to an informed individual who solved the task once per minute, was 7.76 times more likely to solve the task socially than asocially. We converted this measure into the predicted proportion of task solutions that occurred by social transmission (see [20] for details of the conversion). We estimated that 59.7% (57.1–66.6%) of the first task solutions occurred by social transmission in subadults. When viewed together with the positive relationships between affiliative interaction and observation networks, the OADA results suggest that selective observation of affiliates determined the pathways of transmission in this group.

### 3.5. Transmission of task solving in juveniles

OADA in juveniles revealed that the affiliative interaction network and the proximity network provide support for social transmission (support ratios: affiliative = 6.82, proximity = 6.85, table 2). The proximity network included two components: sitting close and sitting on the same branch. The sitting close component without the transmission weights provided the main support (support ratio = 61.48). However, the social transmission rate per unit connection was very low (*s* = 1.08 × 10^−8^), suggesting that other factors besides social connections in these networks better predicted the task-solving order.

Social rank had a strong effect on the task-solving order in juveniles (total Akaike weight for social rank = 95.80%). Juvenile ravens were 2.6 times more likely to solve the task with each increase in rank (95% CI = [1.3, 8.3]). Yet, rank alone was not sufficient to fully explain the transmission patterns because females solved the task 27.3 times sooner than males of the same rank (95% CI = [1.09, 2618]). For instance, the first two solvers were the two dominant males in the group, but they were also siblings of the trained female. The next four solvers (two males, two females) were also siblings of each other. The two females from this sibling group had lower social rank (rank 7 and 8) than the ravens who solved the task later (ranks 5, 6, 10; ravens ranking 6 and 10 are the two juvenile males to whom we presented the task separately from others).

These patterns prompted us to explore the potential role of kinship in transmission. We constructed a kinship network by assigning a connection of 1 between the siblings, and a connection of 0 between the non-siblings. An OADA model based on the kinship network was better supported than the asocial model which included social rank and sex (kinship network AIC_c_ = 15.04, support ratio = 55.4; asocial model AIC_c_ = 23.06). Besides playing a role in the transmission patterns, kinship was also a strong predictor of the affiliative interactions between juveniles (MRQAP, dependent matrix: affiliative network; independent matrices: kinship *r* = 0.638, *p* < 0.001, sex *r* = −0.045, *p* = 0.364, social rank *r* = 0.015, *p* = 0.219). Notably, juveniles initiated their most frequent affiliative interactions towards one of their siblings (figure 2; electronic supplementary material, table S1). Overall, transmission in the juvenile group was predicted by a combination of social rank and the kinship network, which also strongly influenced juveniles' affiliative interactions.

## 4. Discussion

We demonstrate positive relationships between social connections, observation patterns and information spread in two raven groups. Networks based on affiliative interactions and physical proximity were positively correlated with an observation network based on who attended to whose task-solving behaviour, demonstrating that ravens observed their affiliates with whom they shared positive social connections (i.e. affiliative physical contact, food sharing and tolerance of close physical proximity). Information spread was best predicted by social transmission through the affiliative interaction network in the subadults, and by a combination of social rank and social transmission through the kinship network (which influenced affiliative interactions) in juveniles. In particular, ravens with high social centrality solved the task sooner than their less central conspecifics, which resulted in them being central in the observation network due to being observed frequently. Together, these results demonstrate the importance of accounting for multiple types of social connections and attributes (e.g. age, sex, rank, kinship) when investigating spread of information in groups.

### 4.1. Observation networks are a valuable tool in transmission studies

The robust positive relationships between networks based on observation and networks based on social connections provide empirical evidence that observation networks are a valuable tool in transmission studies. Observation can play at least two roles in information transmission. First, observing conspecifics interact with a novel task or a novel object may decrease neophobia and increase interest in the task or the object. This effect may be especially pronounced in species with high neophobia, such as ravens. Naive ravens were more likely to interact with the novel task after observing informed conspecifics interact with it, a pattern that is also documented in meerkats [25] and squirrel monkeys [19]. Second, naive individuals may observe informed conspecifics to learn the association between their behaviour and the outcome, for which repeated observations from a close distance may be necessary [54–56]. Such repeated instances of observation can only be achieved if the observer(s) and the observed individual share positive social connections, allowing them to tolerate each other in close proximity. In our study, networks based on affiliative interactions and physical proximity were the most reliable predictors of who observed whom. Similarity in sex or social rank was not influential in ravens' decision of whom to observe, suggesting that group members of different sexes or social ranks can observe each other if they share positive social connections.

We suggest that more information transmission studies should utilize observation networks when assessing the relationships between social connections and information acquisition. Our observation networks included only the group members who were observing within 1 m of the task. This was necessary because multiple ravens were present around the task during the trials, possibly preventing those who were farther away from the task from seeing the solution technique. However, it is possible that ravens may have observed from a distance, especially during the trials in which only a few ravens were present around the task. Future research on information transmission should account for the possibility that conspecifics may acquire information from others by observing from a distance, as has been shown in New Caledonian crows [57].

### 4.2. Quantifying multiple social connections is essential for understanding observation and transmission

Not all social connections were equally effective at predicting the patterns of selective observation and information spread. In subadults, only the affiliative interaction network, but not the proximity network nor the aggressive interaction network, provided support for social transmission against asocial learning. Furthermore, there was considerable variation in how reliably different types of affiliative behaviours predicted transmission. For example, the affiliative network included two components (physical contact such as allo-preening, and food/object sharing), and the main support for transmission came from the physical contact component. Allo-grooming (and allo-preening) is one of the main forms of social bonding in animals, and the dyads with the strongest social bonds tend to groom each other more frequently than the dyads with weak or no bonds [35]. Such strong positive social bonds would allow conspecifics to tolerate each other in close proximity, motivating them to observe each other's task-solving behaviour to acquire information about the task, which they would then use to solve the task. For example, ravens with high affiliative network centrality in both groups solved the task sooner, possibly because they were connected to at least one informed conspecific whom they could repeatedly observe from a close distance. These central ravens were then observed more by naive conspecifics, and thus had high centrality in the observation networks, leading to strong relationships between affiliative interaction networks, observation networks and information transmission.

Studies on information transmission will greatly benefit from including multiple networks based on different types of social connections. However, in doing so, it will be critical to ensure that the social connection data are collected independently of the novel information data. The presence of resources (e.g. a novel task) may bias associations and social interactions, causing individuals to associate or interact with the conspecifics with whom they may not have associated or interacted otherwise. As a result, network data obtained in the presence of a task may not be representative of the true social connections between conspecifics. We avoided this issue by obtaining social connection data (i.e. interaction and proximity network data) only during the days in which we did not run the task trials. We strongly suggest that the potential confounding effects of task presence on social data are kept in mind during group transmission studies, particularly when analysing the relationships between social transmission patterns and the social networks that are obtained in the presence of the novel information of interest.

### 4.3. Group composition influences transmission patterns

The role of social connections in information acquisition and transmission may change due to differences in group composition and structure, especially in species that face frequent changes in group dynamics. Individuals living in fission–fusion groups, such as wild non-breeding ravens [30,31], frequently have to deal with changing group dynamics. Although the captive groups we studied did not experience fission–fusion dynamics, because they differed in age (subadult versus juvenile) and kinship, we were able to explore the influence of age and kinship variation on transmission. In subadults, selective observation of affiliates determined the task-solving order and the pathways of information transmission. However, in comparison with the subadult group, evidence for social transmission through affiliative networks was not as robust in the juvenile group, as indicated by the low rates of social transmission per unit connection. Instead, a combination of social rank and kinship network predicted the task-solving order in juveniles. After a raven had solved the task, the next group members to solve were his siblings, starting with the most dominant sibling. It is possible that different sibling groups gained access to the task at different times. As a result, the order of access to the task, both within and between sibling groups, may have played a role in the pathways of transmission in juveniles.

Even though the role of affiliative interactions in information spread was not as clear in juveniles as it was in subadults, affiliation may have had an indirect influence in this group. The affiliative interaction network of juveniles had higher density than that of the subadults, and juveniles shared affiliative interactions with more group members than subadults did. Yet, despite the highly connected nature of the affiliative network, there was also evidence of social selectivity in juveniles' affiliative interactions with each other. Juveniles' most frequent affiliative interactions, which indicate strong social bonds, were with their siblings. In comparison, subadults' strongest bonds were generally within the male–female pairs. In both groups, frequent affiliative interactions predicted who observed whom most frequently. In juveniles, the strong social bonds between the siblings may have played an important role in transmission, allowing them to observe and learn from the siblings with whom they shared their strongest social bonds.

## 5. Conclusion

By constructing networks on multiple social connections, and by integrating network analysis with information transmission experiments, we show that network analysis can be used to assess the patterns of selective observation and information transmission. Observation networks are rarely used in transmission studies, but they provide critical insights into understanding the relationships between social connections and spread of information. Yet, not all social connections are equally effective at influencing the patterns of observation and transmission. Connections based on positive social behaviours, such as affiliative interactions and tolerance of close physical proximity, can be more informative than other social connections. Furthermore, group differences may also play a major role in transmission. In some groups, networks based on individual attributes (e.g. age, sex, kinship) may be better predictors of information transmission patterns than networks based on social connections. Therefore, it is critical to account for multiple types of networks to achieve a comprehensive understanding of information transmission in groups.

## Supplementary Material

Kulahci et al. List of supplementary information. Kulahci et al.

## Supplementary Material

Supplementary Methods: Explanation of Network Based Diffusion Analysis (NBDA) and Order of Acquisition Diffusion Analysis (OADA). Kulahci et al.

## Supplementary Material

Supplementary Tables: Tables S1-S3. Kulahci et al.

## Data accessibility

The data supporting this article are included as part of the electronic supplementary material.
