# Supplementary material for: Social networks predict selective observation and information spread in ravens
Source: R Soc Open Sci. 2016 Jul 13;3(7):160256. doi: 10.1098/rsos.160256 (PMC4968472; doi:10.1098/rsos.160256)
Supplement: Kulahci et al. List of supplementary information. Kulahci et al. [file rsos160256supp1.pdf]

List of Supplementary Information

Social networks predict selective observation
and information spread in ravens

Ipek G. Kulahci, Daniel I. Rubenstein, Thomas Bugnyar,
William Hoppitt, Nace Mikus & Christine Schwab

Supplementary Methods:

Explanation of Network Based Diffusion Analysis (NBDA) and Order of Acquisition Diffusion Analysis (OADA).

Supplementary Tables:

Table S1. Individual attributes and task data.

Table S2. Ranked network centrality metric data.

Table S3. Relationships between social centrality, task solving, and observation centrality.

Supplementary Videos:

Video S1. A raven solves the task while another raven observes. The two steps to get the reward are clearly visible in the video. The first step involves opening the Velcro, the second step involves pulling open a drawer.

Video S2. Multiple ravens were present around the task during a typical trial. Because presence of multiple ravens around the task may prevent other group members from seeing the solution technique from a distance, we defined observers as those within 1m radius of the task.
