# Supplementary material for: Social networks predict selective observation and information spread in ravens
Source: R Soc Open Sci. 2016 Jul 13;3(7):160256. doi: 10.1098/rsos.160256 (PMC4968472; doi:10.1098/rsos.160256)
Supplement: Supplementary Methods: Explanation of Network Based Diffusion Analysis (NBDA) and Order of Acquisition Diffusion Analysis (OADA). Kulahci et al. [file rsos160256supp2.pdf]

Social networks predict selective observation and information spread in ravens (Kulahci, I.G., Rubenstein, D.I., Bugnyar, T., Hoppitt, W., Mikus, N. & Schwab, C.)

Supplementary Methods: Explanation of Network Based Diffusion Analysis (NBDA) and Order of Acquisition Diffusion Analysis (OADA)

In the standard NBDA model, the rate at which individual *i* acquires the novel behaviour (e.g. solves a task; discovers a food patch) at time *t* is given by:

$$\lambda_i(t) = \lambda_0(t)\left(s\sum_{j=1}^{N} a_{ij}z_j(t) + 1\right)\left(1 - z_i(t)\right)$$

where $\lambda_0(t)$ is a baseline rate function (see below), a_{ij} is the network connection from *j* to *i*, $z_i(t)$ is the status (0= naïve; 1= informed) of *i* at time *t*, *N* is the number of individuals in the diffusion and *s* is a parameter giving the rate of social transmission (Hoppitt, Boogert & Laland, 2010).

NBDA can include other individual-level variables in a **multiplicative model**:

$$\lambda_i(t) = \lambda_0(t)\left(s\sum_{j=1}^{N} a_{ij}z_j(t) + 1\right)exp(LP_i)\left(1 - z_i(t)\right)$$

or **an additive model:**

$$\lambda_i(t) = \lambda_0(t)\left(s\sum_{j=1}^{N} a_{ij}z_j(t) + exp(LP_i)\right)\left(1 - z_i(t)\right)$$

where LP_i is a linear predictor combining *V* variables analogous to a generalized linear model:

$$LP_i = \sum_{1}^{V} \beta_l x_{li}$$

where x_{li} is the value of the lth individual level variable for individual *i*, and $exp(\beta_l)$ gives the multiplicative effect of one unit increase in that variable.

The model can further be expanded by taking transmission weights (w_j) into account:

$$\lambda_i(t) = \lambda_0(t)\left(s\sum_{j=1}^{N} a_{ij}w_jz_j(t) + 1\right)\left(1 - z_i(t)\right)$$

where w_j is a measure of how often individual *j* solved the task after learning.

There are various version of NBDA: order of acquisition diffusion analysis (OADA, Hoppitt, Boogert & Laland, 2010), discrete time of acquisition diffusion analysis (discrete TADA; Franz & Nunn, 2009), and continuous TADA (Hoppitt, Boogert & Laland, 2010). In this paper, we used the OADA variant since it makes no assumptions about the shape of the underlying distribution of latencies.

In OADA, the likelihood for each solving event is given by:

$$\lambda_{SOLVER}(T) \Big/ \sum_{1}^{N} \lambda_i(T)$$

where T is the time at which the solving event occurred and $\lambda_{SOLVER}(T)$ is the rate of solving for the solver at that time. Note that the baseline learning rate, $\lambda_0(T)$ cancels outs in the numerator and denominator with the assumption that it is the same for all individuals. We summed the log likelihoods across events to get the total log-likelihood.

We used an information-theoretic approach using corrected Akaike's Information Criterion (AIC_c) (Burnham & Anderson, 2002). We calculated Akaike weights giving the support for each specific model, allowing us to assess the support for each network (see Tables 3, S2, S3; see below for more information). We fitted all models in the R statistical environment version 2.15.2 (R Core Team, 2012), using the optim function.

We obtained model-averaged estimates for all parameters (Burnham & Anderson, 2002). We used the numerical estimate of the Hessian matrix (Morgan, 2008) to estimate standard errors, allowing us to calculate an unconditional standard error to account for model selection uncertainty (Burnham & Anderson, 2002). To quantify the uncertainty in estimates for key parameters, we obtained 95% confidence intervals using the profile likelihood technique, expanded to allow for model selection uncertainty (Burnham & Anderson 2002).

"s" parameters for unweighted networks can be interpreted as the rate of solving by social transmission per unit of association to informed individuals, relative to the baseline rate of asocial learning- i.e. when the linear predictor, LP_i, is set to zero. For weighted networks, s is the rate of solving by social transmission (per unit of association to informed individuals x unit of weighting), relative to the baseline rate of asocial learning. To give a more intuitive measure of the importance of each type of social transmission, we calculated the probability each event occurred as a result of each type of social transmission (extending the method used by Allen et al., 2013):

$$p_i = s\sum_{j=1}^{N} a_{ij} z_j(T) \Bigg/ \left(s\sum_{j=1}^{N} a_{ij} z_j(T) + exp(LP_i) \right)$$

where *i* is the individual solving the task and *T* is the time at which task solution occurred. We added these figures across all solves (excluding the trained individuals) to get an estimate of the proportion of events that occurred by asocial learning and by social transmission.

In subadults, the results of multiplicative and additive models were similar. Thus, we presented the results of the multiplicative model, which had slightly more support than the additive model (1.44x). In the juveniles, we presented the results of the additive model, which had more support (14.7x) than the multiplicative model.

Calculation of support ratios

Instead of using model-selection to choose a best predictive model, we used a model averaging approach, using Akaike's information criterion, corrected for sample size (AICc) (Burnham and Anderson, 2002). Model averaging takes into account uncertainty as to which model is best. AICc estimates the Kullback – Leibler (K – L) information for a model (how well the predicted distribution for the dependent variable approximates its true distribution). The AICc allowed us to calculate an Akaike weight for each model giving the probability that the model is the actual best model, i.e. has the lowest K – L information, out of all those considered, allowing for sampling variation.

By summing Akaike weights for all models that include a specific social network, we obtained the probability that a social network or other variable is in the best K – L model, thus quantifying the support the data give for social transmission following that network. To standardize the support for each social network, such that they quantify the support for each network relative to the support for asocial learning, we divided the total Akaike weight for each by the total Akaike weight for asocial learning. We refer to these ratios as 'support ratios'.

To help scale the reader's interpretation of the strength of these support ratios, we note that a p-value of 5% in a likelihood ratio test between models differing in one parameter (e.g. social transmission via a network) corresponds to a support ratio of 2.5.

REFERENCES:

Allen J, Weinrich M, Hoppitt W, & Rendell L (2013) Network-based diffusion analysis reveals cultural transmission of lobtail feeding in humpback whales. *Science* **340**:485-488.

Burnham, KP & Anderson, DR (2002) Model selection and multimodel Inference: A practical information-theoretic approach. 2nd edition. Springer.

Hoppitt W & Laland KN (2013) *Social Learning: An Introduction to Mechanisms, Methods and Models*. Princeton University Press.

Hoppitt W, Kandler A, Kendal JR & Laland KN (2010) The effect of task structure on diffusion dynamics: Implications for diffusion curve and network-based analyses. *Learning & Behavior* **38**: 243-251.

Morgan, BJT (2008) *Applied Stochastic Modelling*. 2nd Edition. CRC Press.

R Core Team (2012) *R: A language and environment for statistical computing.* R Foundation for Statistical Computing, Vienna, Austria. http://www.R-project.org/
